# Disseminated Bacillus Calmette-Guérin (BCG) Infection Presenting With a Miliary Pulmonary Pattern Following Intravesical Therapy

**DOI:** 10.7759/cureus.105658

**Published:** 2026-03-22

**Authors:** Daniela Baptista, Filipe Dias, Sofia Sobral, Rui Dinis, Henrique Rita

**Affiliations:** 1 Internal Medicine, Unidade Local de Saúde de Lisboa Ocidental, Lisbon, PRT; 2 Internal Medicine, Unidade Local de Saúde do Litoral Alentejano, Santiago do Cacém, PRT; 3 Urology, Unidade Local de Saúde do Litoral Alentejano, Santiago do Cacém, PRT

**Keywords:** bacillus calmette-guerin, bladder carcinoma, disseminated infection, intravesical therapy, mycobacterium bovis

## Abstract

Intravesical administration of Bacillus Calmette-Guérin (BCG) is a commonly employed therapeutic approach in clinical practice for the treatment of bladder neoplasms. Although it is generally safe, severe systemic complications may rarely arise. This report describes a patient with non-invasive papillary urothelial carcinoma treated with intravesical BCG therapy who developed disseminated infection with a miliary pattern. Although multiple attempts were made to isolate *Mycobacterium bovis* from blood, urine, and sputum samples, microbiological confirmation was never obtained. The diagnosis was therefore established presumptively based on the clinical presentation and epidemiological context, and anti-mycobacterial therapy was initiated accordingly. The patient’s favorable clinical response to treatment further supported the diagnosis. This report describes a rare complication and a challenging diagnosis that should be considered in patients undergoing this form of treatment.

## Introduction

Intravesical administration of Bacillus Calmette-Guérin (BCG), an attenuated strain of *Mycobacterium bovis*, is an adjuvant therapy for bladder carcinoma associated with reduced recurrence rates and a lower risk of progression following transurethral resection of bladder tumor (TURBT). Infectious complications occur in approximately 1-5% of cases [1-4]. Complications may present as localized genitourinary symptoms such as dysuria, urinary frequency, and hematuria. In rare cases, systemic dissemination may occur with hepatic, pulmonary, or articular involvement, miliary disease, or sepsis [1,5,6].

Systemic BCG infection should be suspected in patients treated with intravesical BCG who develop systemic manifestations, particularly within the first three months following instillation. Risk factors for this complication include catheterization, active cystitis, persistent macroscopic hematuria following transurethral surgery, immunosuppression, and age ≥70 years [7].

In this study, we present a rare and clinically significant case of disseminated BCG infection following intravesical administration for recurrent bladder neoplasia. This case is particularly remarkable due to the absence of microbiological isolation, which made the diagnosis challenging and required careful clinical and radiological correlation. The constellation of findings and the temporal relationship with BCG therapy strongly supported the diagnosis, underlining the diagnostic complexity and the need for clinical vigilance in similar presentations.

## Case presentation

We present a case of a 69-year-old independent male, former smoker (60 pack-years), with a medical history of essential hypertension, dyslipidemia, and non-invasive papillary urothelial carcinoma approximately 20 years earlier. The carcinoma was treated with transurethral resection of bladder tumor (TURBT) and intravesical mitomycin C. He remained under ultrasound surveillance without recurrence until early 2024, when a new bladder nodule was detected. He underwent cystoscopy and repeated TURBT, with histopathological confirmation of recurrence.

Intravesical BCG therapy was initiated and administered over six months (12 instillations) without immediate complications. Subsequently, the patient developed hematuria and underwent repeat cystoscopy, which revealed a new bladder lesion. A second course of BCG therapy was initiated. Following the first instillation (of the second course of treatment), the patient developed severe dysuria and marked urinary frequency, particularly nocturnally. A local inflammatory reaction was initially assumed, and treatment with flavoxate, ibuprofen, and tamsulosin was prescribed without improvement. A urinary tract infection was presumed, a urine culture was obtained, and prulifloxacin 600 mg once daily was initiated for 10 days. The culture subsequently yielded negative results. Two weeks later, after the second BCG instillation, he developed evening pyrexia (maximum axillary temperature 39.4°C), nocturnal diaphoresis, malaise, and fatigue. Dysuria and frequency persisted, but he denied dyspnea, cough, arthralgia, abdominal pain, diarrhea, or neurological symptoms.

Symptoms progressed over two more weeks, and his general practitioner requested an abdominopelvic CT scan and referred him to the emergency department. On assessment, he was alert, oriented, and hemodynamically stable, though febrile (38.4°C) and reporting marked fatigue. Neurological and respiratory examinations were unremarkable, and chest auscultation was normal. Laboratory findings showed elevated inflammatory markers, transaminases, gamma-glutamyl transferase, and adenosine deaminase (ADA) (Table 1).

**Table 1:** Laboratory results at hospital admission.

Analysis	Result	Reference range
Hemoglobin	14.8 g/dL	13.5-17.2 g/dL
Leukocytes	7.1x10³/µL	4.0-10.0x10³/µL
Neutrophils	75.2%	40-70%
Lymphocytes	14.9%	20-40%
Platelets	266,000 cells/µL	150,000-400,000 cells/µL
Urea	53 mg/dL	15-45 mg/dL
Creatinine	1.1 mg/dL	0.7-1.2 mg/dL
Sodium (Na)	130 mEq/L	135-145 mEq/L
Potassium (K)	4.0 mEq/L	3.5-5.0 mEq/L
Chlorine (Cl)	91 mEq/L	98-106 mEq/L
Lactate dehydrogenase (LDH)	277 U/L	125-220 U/L
Aspartate aminotransferase (AST)	82 U/L	15-37 U/L
Alanine aminotransferase (ALT)	125 U/L	10-40 U/L
Total bilirubin (BT)	0.7 mg/dL	0.1-1.2 mg/dL
Direct bilirubin (BD)	0.27 mg/dL	0-0.3 mg/dL
Gamma-glutamyl transferase (GGT)	479 U/L	10-71 U/L
Alkaline phosphatase (ALP)	151 U/L	44-147 U/L
Adenosine deaminase (ADA)	38.9 U/L	0-19 U/L
Erythrocyte sedimentation rate (ESR)	67 mm/h	0-15 mm/h
C-reactive protein (CRP)	7.91 mg/dL	0-5 mg/dL
Procalcitonin	0.86 ng/mL	0.05-0.5 ng/mL

**Figure 1:**
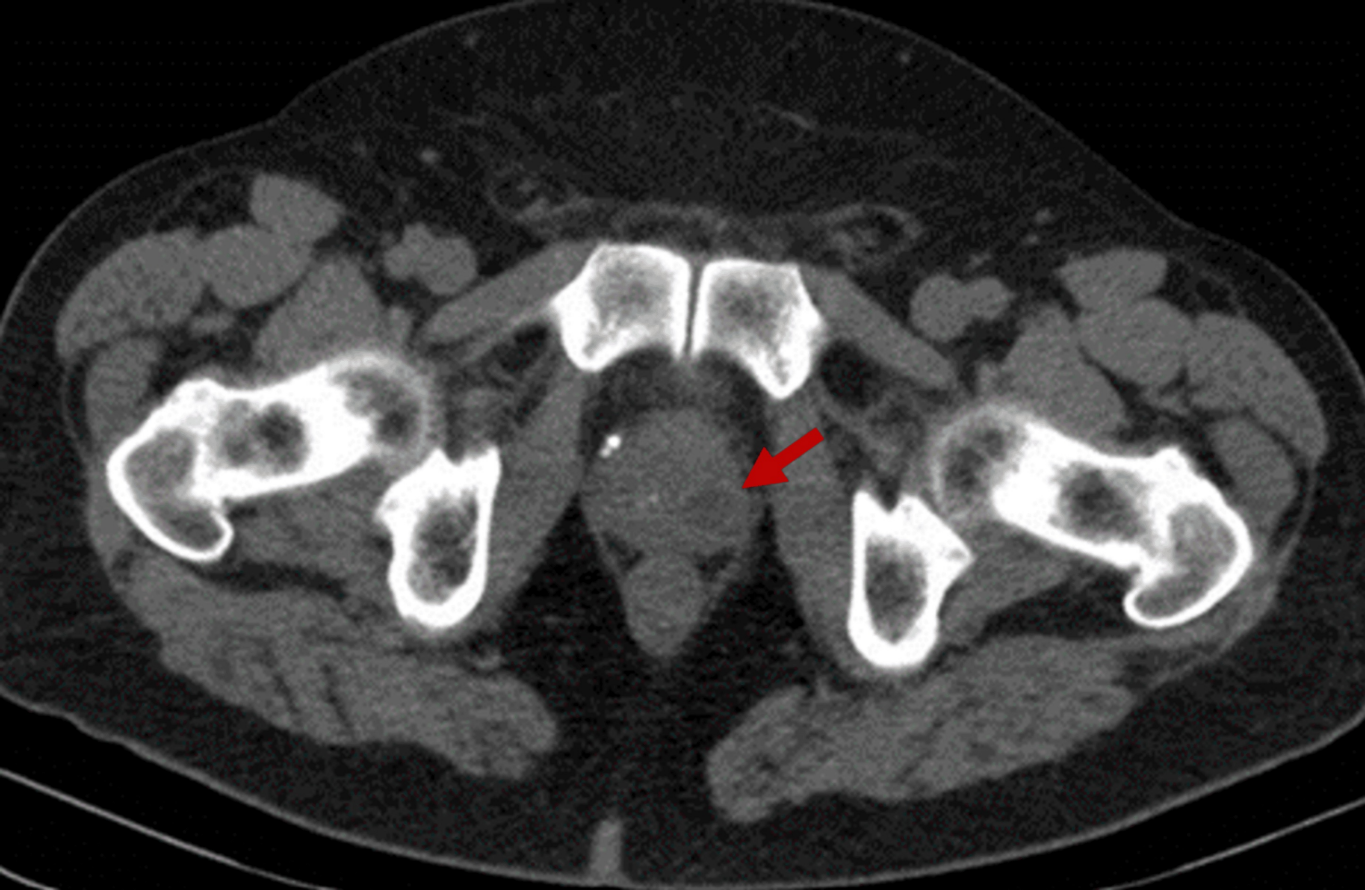
Abdominopelvic CT scan demonstrating a fluid-density lesion in the left transition zone of the prostate suggestive of a prostatic abscess (arrow).

**Figure 2:**
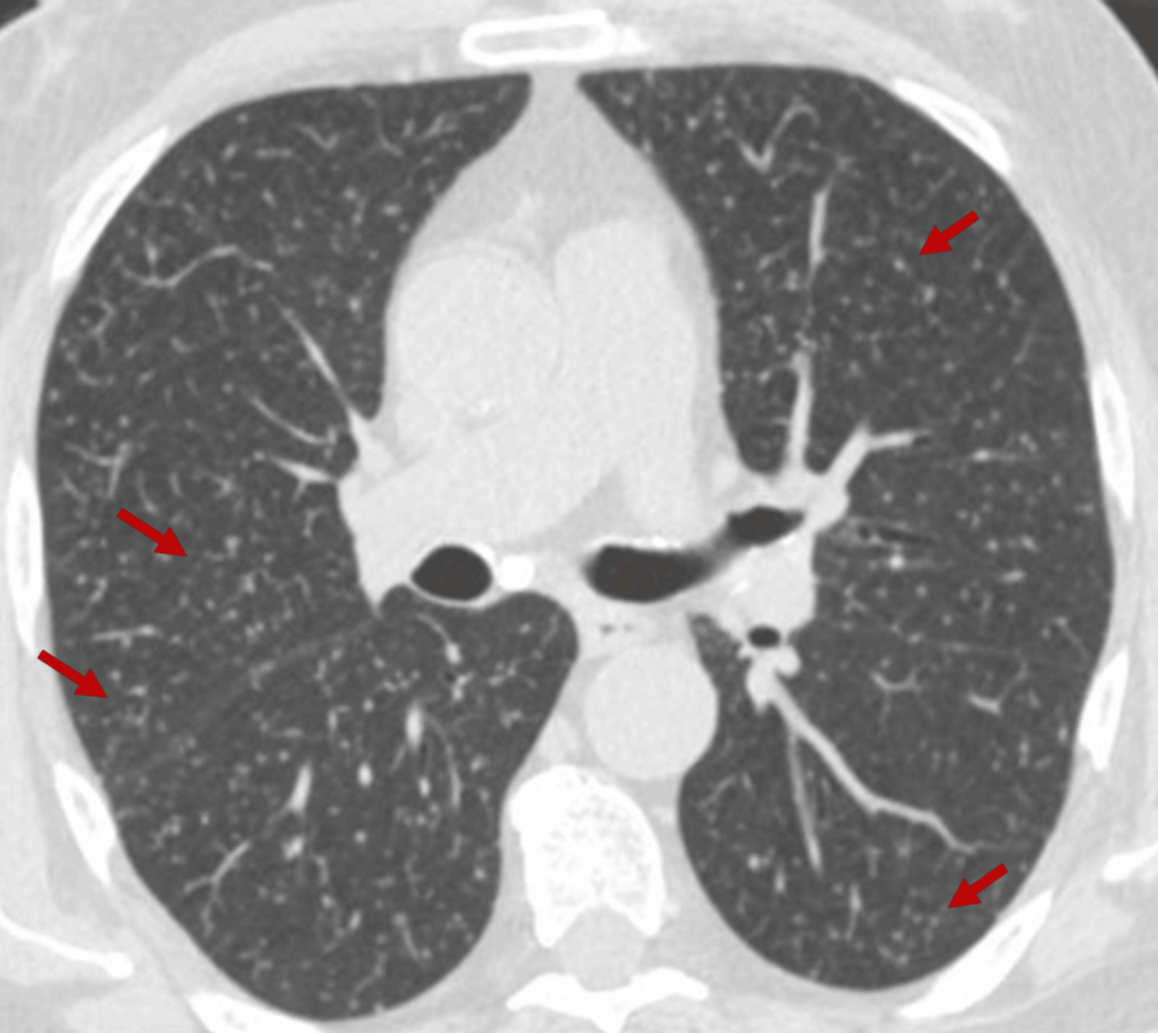
Chest CT showing diffuse bilateral micronodular infiltration in a miliary pattern (arrows).

Blood cultures, urine culture, and mycobacterial cultures were requested. Abdominopelvic CT scan demonstrated focal thickening of the anterior bladder wall (cystitis not excluded), a 15 mm fluid-density lesion in the left transition zone of the prostate, suggestive of prostatitis or possible abscess (Figure 1). A chest CT scan was also performed, which showed diffuse bilateral micronodular infiltration with a miliary pattern, possibly indicating an infectious process or disseminated metastases, requiring correlation with clinical and laboratory findings (Figure 2).

The patient was admitted for further investigation. Disseminated BCG infection was presumed in the context of intravesical therapy for bladder carcinoma, with bladder, prostatic (with abscess), pulmonary, and hepatic involvement. Anti-tuberculous therapy was initiated with rifampicin 600 mg/day, isoniazid 300 mg/day, and ethambutol 1200 mg/day, with pyridoxine 40 mg/day supplementation. During hospitalization, he remained hemodynamically stable but experienced multiple daily febrile spikes (maximum 39.9°C), without respiratory compromise, developing only a dry cough. Blood cultures, urine culture, acid-fast bacilli (AFB) smear, and nucleic acid amplification testing (NAAT) in urine were negative. Mycobacterial cultures were also negative. Bronchoscopy was performed, but direct examination, NAAT, and cultures were also negative.

Due to persistent high-grade fever and following discussion with urology, transrectal drainage of the prostatic abscess was undertaken. *Staphylococcus haemolyticus*, sensitive only to vancomycin and gentamicin, was isolated, and linezolid was administered for 14 days. Follow-up imaging showed persistence of the small abscess, unchanged in size. Transthoracic echocardiography showed no evidence of endocarditis.

After approximately one month of anti-tuberculous therapy, the fever pattern improved, becoming less frequent and lower-grade, which was interpreted as a favorable therapeutic response. Clinical improvement was accompanied by normalization of inflammatory markers. The prostatic abscess was considered part of the disseminated BCG infection and was managed conservatively due to its small size.

On day 43 of anti-tuberculous therapy, the patient was hemodynamically stable, without respiratory complaints, with improvement in genitourinary symptoms and appetite. He continued to experience a single febrile episode (maximum 38°C) approximately every four days. Given overall clinical and laboratory improvement, he was discharged with follow-up in urology and a tuberculosis clinic. At the one-month review, he was afebrile with stable laboratory findings. He underwent a complete TURBT, and histology revealed chronic ulcerative cystitis, possibly iatrogenic in etiology, with no evidence of cellular atypia.

## Discussion

Infectious complications following BCG intravesical therapy are estimated to occur in approximately 1-5% of cases [1-4]. The risk of BCG infection is highest in the first two years following treatment initiation, and more serious systemic infections, such as those affecting the lungs and liver, tend to manifest earlier [4]. The mechanism of BCG dissemination is generally through the urinary tract and potentially hematogenously. A key factor in localized spread is male anatomy, which may predispose to intraductal spread through the prostate (especially after TURBT) or along the vas deferens to the epididymis and testis. Additionally, male anatomy increases the risk of traumatic catheterization during instillation, potentially facilitating entry of BCG into the bloodstream or tissues [4].

Disseminated BCG often presents with non-specific clinical features, making early recognition challenging. Persistent fever, weight loss, elevated inflammatory markers, and liver function abnormalities should raise suspicion, particularly after recent intravesical BCG instillation. Negative cultures do not exclude diagnosis due to the typically low mycobacterial load and prolonged culture times [8,9].

In this case, pre-existing local inflammation and repeated instillations may have facilitated systemic dissemination. The histopathological finding of ulcerative cystitis supports this mechanism. The clinical presentation, imaging findings, and temporal relationship with bladder instillations guided the diagnosis and prompted therapy.

We followed the guidelines and initiated recommended treatment with isoniazid, rifampicin, and ethambutol for two months, followed by seven months of isoniazid and rifampicin, with pyridoxine supplementation to prevent neuropathy. Pyrazinamide was excluded due to *M. bovis* resistance [10].

This case presents some important limitations. The diagnosis was presumptive, based on clinical presentation, imaging findings, and temporal relationship with the instillations, but was not confirmed by microbiological or histological methods. The identification of *Staphylococcus haemolyticus* in the prostatic abscess is a confounding factor, which does not allow us to exclude a mixed or overlapping infection. Additionally, the lack of histological confirmation of the pulmonary and hepatic lesions limits the definitive exclusion of important differential diagnoses, particularly metastatic disease, although the favorable outcome with anti-tuberculous and antibiotic therapy is highly suggestive of the assumed diagnosis.

## Conclusions

Disseminated BCG infection remains a rare but potentially serious complication of intravesical therapy. Diagnosis is often challenging due to non-specific symptoms and the frequent absence of microbiological confirmation, as cultures frequently yield negative results. Therefore, clinicians should maintain a high index of suspicion in any patient presenting with persistent fever, constitutional symptoms, or unexplained laboratory abnormalities, even weeks after the last instillation. Given the low yield of cultures, a negative microbiological result should not delay the initiation of empirical anti-tuberculous therapy when clinical and radiological suspicion is high.

Cross-sectional imaging is valuable for evaluating complications, and a multidisciplinary approach involving internal medicine, urology, and radiology is essential for timely diagnosis and management. Awareness of this rare but serious complication and its potential presentations can help clinicians minimize diagnostic delay and improve patient prognosis.
